# Infiltrating bone marrow mesenchymal stem cells (BM-MSCs) increase prostate cancer cell invasion *via* altering the CCL5/HIF2α/androgen receptor signals

**DOI:** 10.18632/oncotarget.4515

**Published:** 2015-07-27

**Authors:** Jie Luo, Soo Ok Lee, Yun Cui, Rachel Yang, Lei Li, Chawnshang Chang

**Affiliations:** ^1^ Department of Pathology, University of Rochester, Rochester, NY 14642, USA; ^2^ Department of Urology, University of Rochester, Rochester, NY 14642, USA; ^3^ Department of Radiation Oncology, University of Rochester, Rochester, NY 14642, USA; ^4^ Department of Biology, University of Rochester, Rochester, NY 14642, USA; ^5^ The Wilmot Cancer Center, University of Rochester, Rochester, NY 14642, USA; ^6^ Sex Hormone Research Center, Department of Urology, The First Affiliated Hospital, Xian Jiaotong University, Xian 710061, China; ^7^ Sex Hormone Research Center, China Medical University and Hospital, Taichung, 404, Taiwan

**Keywords:** CCL5, bone marrow mesenchymal stem cells, HIF2α, androgen receptor, prostate cancer

## Abstract

Several infiltrating cells in the tumor microenvironment could influence the cancer progression *via* secreting various cytokines. Here, we found the CCL5 secreted from BM-MSCs suppressed androgen receptor (AR) signals *via* enhancing the expression of hypoxia inducible factor 2α (HIF2α) in prostate cancer (PCa) cells. Mechanism dissection revealed that the increased HIF2α might alter the AR-HSP90 interaction to suppress the AR transactivation, and inhibition of HIF2α reversed the BM-MSCs-increased PCa stem cell population and PCa cells invasion. Importantly, CCL5 could suppress the prolyl hydroxylases (PHDs) expression, which might then lead to suppress VHL-mediated HIF2α ubiquitination. Together, these results demonstrated that the CCL5 signals from infiltrating BM-MSC cells to HIF2α signals within PCa cells might play a key role to increase PCa stem cell population and PCa metastasis *via* altering the AR signals. Targeting this newly identified CCL5/HIF2α/AR axis signal axis may allow us to develop a novel way to suppress PCa metastasis.

## INTRODUCTION

Recent studies suggested that several cell types in the prostate tumor microenvironment (TME) might contribute to the prostate cancer (PCa) progression [1–11]. For example, infiltrating macrophages might promote PCa metastasis *via* modulation of CCL2/CCR2-STAT3 signaling [7–9] and recruited endothelial cells might also be able to promote PCa metastasis *via* modulation of IL6 signaling [10]. Our previous study demonstrated that infiltrating bone marrow derived mesenchymal stem cells (BM-MSCs) might be able to enhance PCa cell invasion *via* altering the cancer stem cell differentiation. The mechanism dissection revealed that this regulation involves the modulation of CCL5 and AR signaling [11]. The CCL5 secreted from BM-MSCs can increase the cancer stem cell and EMT markers, such as the CD133, ZEB-1 and CXCR4.

The findings that AR in individual cells within the TME might play differential roles (positive *vs* negative roles) could further complicate the androgen/AR signaling in PCa progression [7–13] and raised special questions about the current androgen deprivation therapy (ADT), which systematically suppresses/reduces androgen from binding to AR in every cell, to suppress the progression of PCa, a disease that has become the most prevalent cancer among males in United States with the 2nd highest mortality rate. [8, 9, 14, 15]. Therefore, better understanding the differential AR signaling in each cell within the TME and from those distinct AR signals to develop better target(s) to modulate AR-mediated PCa in selective cells may help us to battle PCa in future.

Hypoxia-inducible factor (HIF) is the central component in response to hypoxia in the cell. Substantial studies reveal that HIF is important for the tumor growth and metastasis [16]. Under hypoxia conditions, the HIF expression can be immediately regulated by the proteasome pathway. The prolyl hydroxylases (PHDs), members of the iron- and 2-oxoglutarate-dependent dioxygenase enzyme family, can be suppressed by hypoxia. The PHDs can hydroxylate the HIFs and then promote the ubiquitination of HIF through its E3 ligase—von Hippel-Lindau tumor suppressor (VHL). There are several genes down stream of HIFs that play an important role in the cancer progression including VEGF [17]. A recent study revealed that the HIFs also regulate the cancer stem cell population [18].

Here we identify that HIF2 α [19–21] may link the signaling between CCL5 and AR to enable the recruited BM-MSCs to promote PCa cell invasion. Mechanism dissection found CCL5 might function through modulation of HIF2α ubiquitination to stabilize the HIF2α protein, and then alter the interaction of HSP90 and AR that resulted in suppression of AR nuclear translocation and AR transactivation.

## RESULTS

### BM-MSCs Increase PCa stem cell population and PCa cell invasion *via* enhancing HIF2α expression

**Figure 1:**
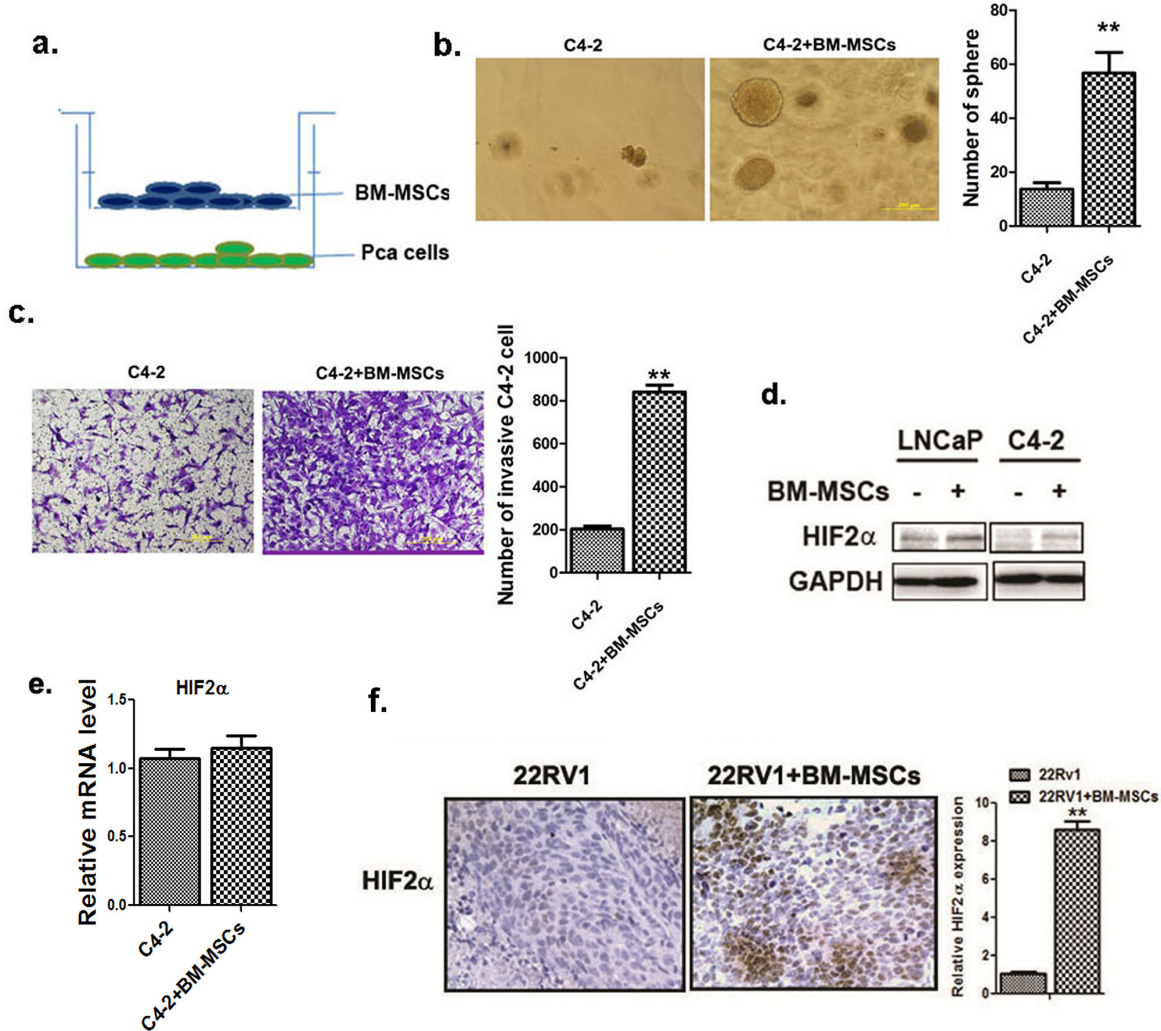
BM-MSCs increase HIF2α expression in PCa cells **a.** The cartoon demonstrating BM-MSCs and PCa cells co-culture 1 × 10^5^ BM-MSCs (with media as control) were placed in upper chambers of the transwell plates (0.4 μm membrane) while PCa cells (1 × 10^6^) were placed in lower chambers. **b.** Sphere formation assay. The PCa cells were co-cultured with the primary MBM-MSCs (media used as control) in 0.4 μM membrane transwell plates for 5 days. Cells were then mixed with Matrigel (1:1, v/v), plated in 24-well plates, and cultured for 10 days. Quantification was shown at right. **c.** Invasion assay result. The C4-2 cells (1 × 10^5^) were co-cultured with the mouse primary BM-MSCs for 3 days in transwell plates (0.4 μM membrane). The invaded cells were stained by toluidine blue, and the positively stained cells were counted from 5 random areas. Quantitation was shown at right. **d.** Western blot analysis of HIF2α expressions. The LNCaP and C4-2 cells were co-cultured with or without the primary mouse BM-MSCs and HIF2α expression was analyzed. **e.** qPCR analysis of HIF2α mRNA level in C4-2 cells with or without BM-MSCs co-culture. **f.** HIF2α IHC staining of the tumor tissues obtained from the CWR22RV1 (22RV1) xenografted mice, with or without co-implantation with the primary BM-MSCs.

We investigated whether the stem cell population in parental PCa cells can be altered after co-cultured with BM-MSCs in a co-culture system as shown in Figure 1a. We observed significant increase in PCa stem cell population when C4-2 cells were co-cultured with BM-MSCs, compared to non-co-cultured condition *via* sphere formation assay (Figure 1b) [22]. We further examined whether this increased stem cell population could influence the invasion ability of PCa cells [23], and results revealed that the invasion ability of these PCa cells are significantly enhanced (Figure 1c). These results confirm our previous report showing the recruited BM-MSCs into PCa led to increase the stem cell population and invasion ability of PCa cells [11].

To dissect the molecular mechanism(s) by which BM-MSCs increase PCa stem cell population, we examined whether BM-MSCs increase stem cell population through activation of several potential signaling pathways that have been reported to be involved in expansion of cancer stem cell population. Among several candidates, we were interested in HIF2α signaling as early studies suggested that HIF2α could induce tumor aggressiveness and expand the cancer stem cell population [24, 25].

We then tested the expression of HIF2α in LNCaP and C4-2 cells after co-culture with BM-MSCs (Figure 1d), and found the increased HIF2α at protein (Figure 1d) and not at mRNA levels (Figure 1e) in these two PCa cells when co-cultured with BM-MSCs.

Similar result were also obtained when we orthotopically xenografted CWR22RV1 PCa cells with BM-MSCs into mouse prostate showing increasing HIF2α expression (Figure 1f).

Together, results from *in vitro* cell lines and *in vivo* mice studies suggest that BM-MSCs may contribute to the increase of the HIF2α expression in PCa cells.

**Figure 2:**
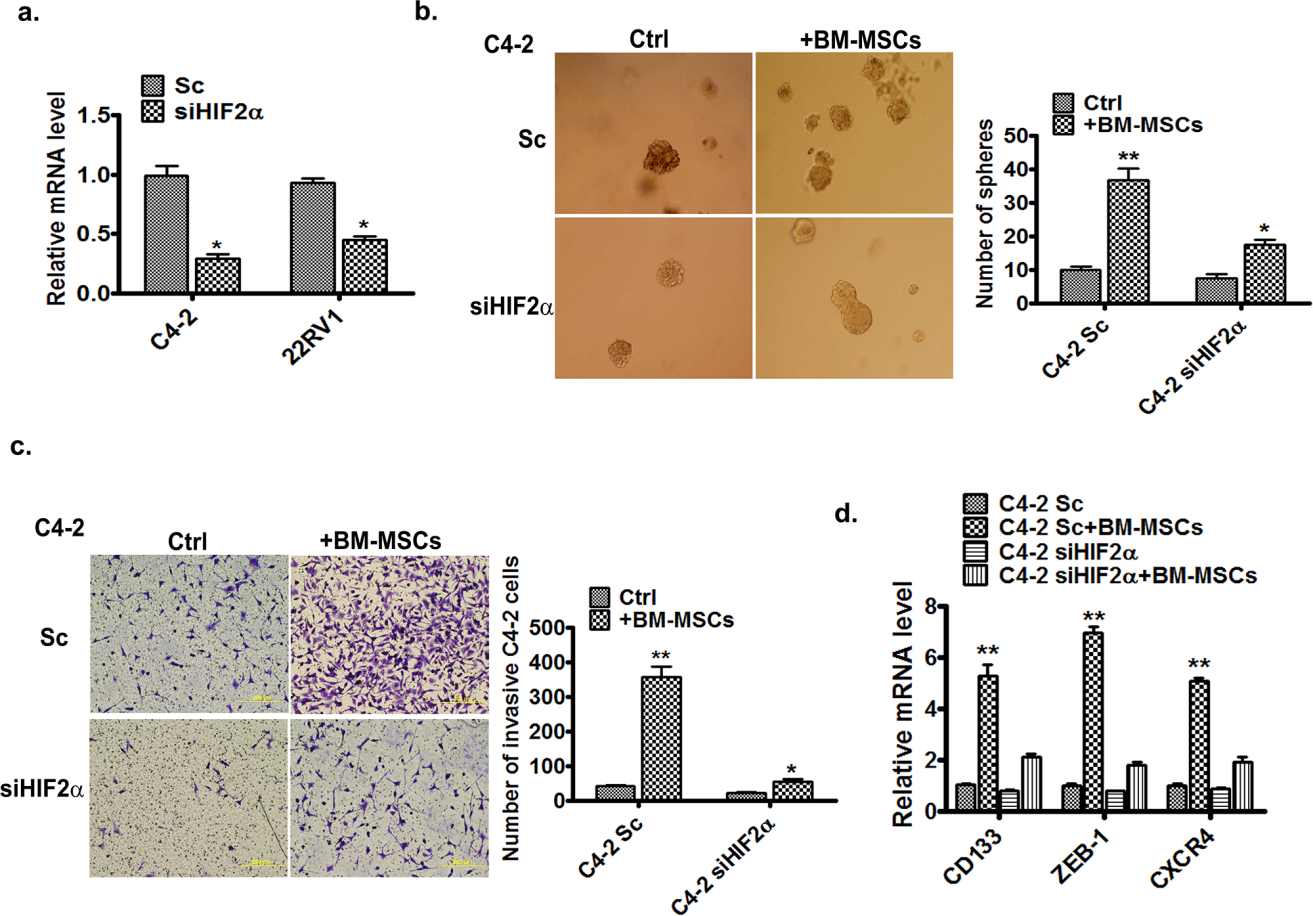
HIF2α is essential for BM-MSCs increase of PCa invasion and stem cell population **a.** qPCR analysis of expression of HIF2α in C4-2 and CWR22RV1 (22RV1) cells after infection by the scramble (Sc) and siHIF2α viruses. **b.** Sphere formation assay of the Sc or siHIF2α C4-2 cells co-cultured with BM-MSCs (media as control). **c.** Invasion assay of the Sc or siHIF2α C4-2 cells co-cultured with the BM-MSCs. **d.** qPCR analysis of expressions of the stem and metastasis marker genes. The Sc or siHIF2α C4-2 cells were co-cultured with or without the primary BM-MSCs. The total RNAs were extracted and the expressions of the CD133, ZEB-1, and CXCR4 genes were analyzed.

To further examine the influences of increased HIF2α on the BM-MSCs-increased PCa stem cell numbers and PCa cell invasion, we applied the interruption approach *via* knocking-down the HIF2α in PCa cells, and results revealed that knocking-down HIF2α (Figure 2a) resulted in suppression of BM-MSCs-enhanced C4-2 stem cell population (Figure 2b) and consequent C4-2 cell invasion (Figure 2c). Importantly, we found knocking-down HIF2α also suppressed those increased stem cell markers (CD133) and metastasis related genes, ZEB-1 and CXCR4 [11, 26] (Figure 2d).

Taken together, results from Figure 1 and 2 suggest that HIF2α may play a key role in mediating the BM-MSCs co-culture effects on the increase of PCa stem cell numbers and PCa cell invasion ability.

### BM-MSCs secrete CCL5 to induce the HIF2α expression

**Figure 3:**
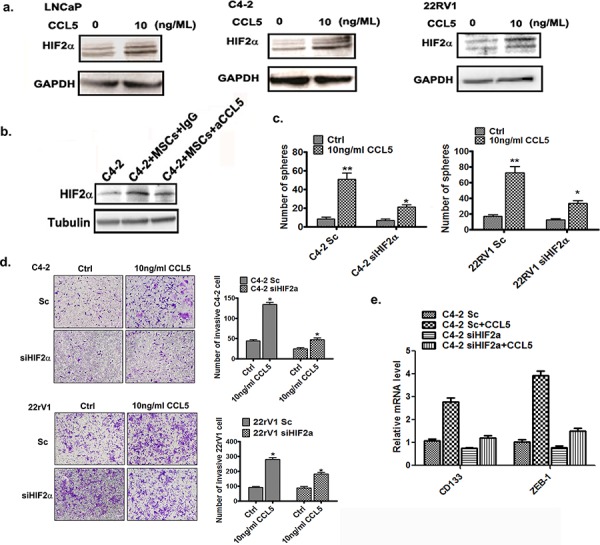
BM-MSCs secrete CCL5 to induce the HIF2α expression **a.** Western blot analysis results of HIF2α expressions after CCL5 treatment. The PCa cells were treated with 10 ng/ML rCCL5 for 48 hrs and expressions of HIF2α were analyzed. **b.** C4-2 cells were co-cultured with BM-MSCs (MSCs) and then treated with either IgG or CCL5 neutralizing antibody. The HIF2α protein levels were then examined by Western blot analysis. **c.** Sphere formation assay of Sc or siHIF2α C4-2 and and CWR22RV1 (22RV1) cells treated by CCL5. The C4-2 and CWR22RV1 cells were infected by scramble or HIF2α siRNA, and then cells were treated with 10 ng/ml CCL5 for 10 days. **d.** Invasion assay of C4-2 and CWR22RV1 cells treated with CCL5. The cells were treated with 10 ng/ml CCL5 for 72 hrs and then the invasion assay was performed. **e.** The qPCR analysis of the mRNA level of CD133 and ZEB-1 in Sc/siHIF2α C4-2 cells treated with 10 ng/ml CCL5.

Next we asked what signaling in BM-MSCs could influence the up-regulation of HIF2α expression in PCa cells upon BM-MScs co-culture. Since early studies indicated that CCL5 is the key cytokine that triggers PCa stem cell increase and PCa cell invasion [11], we were interested to see its impact on the HIF2α expression. By adding the functional CCL5 recombinant protein (rCCL5) directly into the culture of various PCa cell lines, we found increased HIF2α expression in PCa cells (Figure 3a), and blocking CCL5 (*via* CCL5 neutralizing antibody) suppressed the BM-MSCs induced HIF2α expression (Figure 3b).

Together, results from Figure 3a–3b suggest that CCL5 secreted from BM-MSCs may play a key role in modulating the increased expression of HIF2α in PCa cells.

Furthermore, knocking-down HIF2α significantly attenuated the CCL5 effect in increasing PCa stem cell numbers (Figure 3c) and PCa cells invasion (Figure 3d). We also observed the knocking-down HIF2α in PCa cells attenuated the CCL5 effect of up-regulation of CD133 and ZEB-1 (Figure 3e).

Together, results from Figure 3c–3e suggest that the CCL5 effect in enhancing PCa stem cell population and PCa cell invasion is *via* altering the HIF2α signals.

### CCL5 suppresses HIF2α ubiquitination by inhibiting the VHL-HIF2α interaction

**Figure 4:**
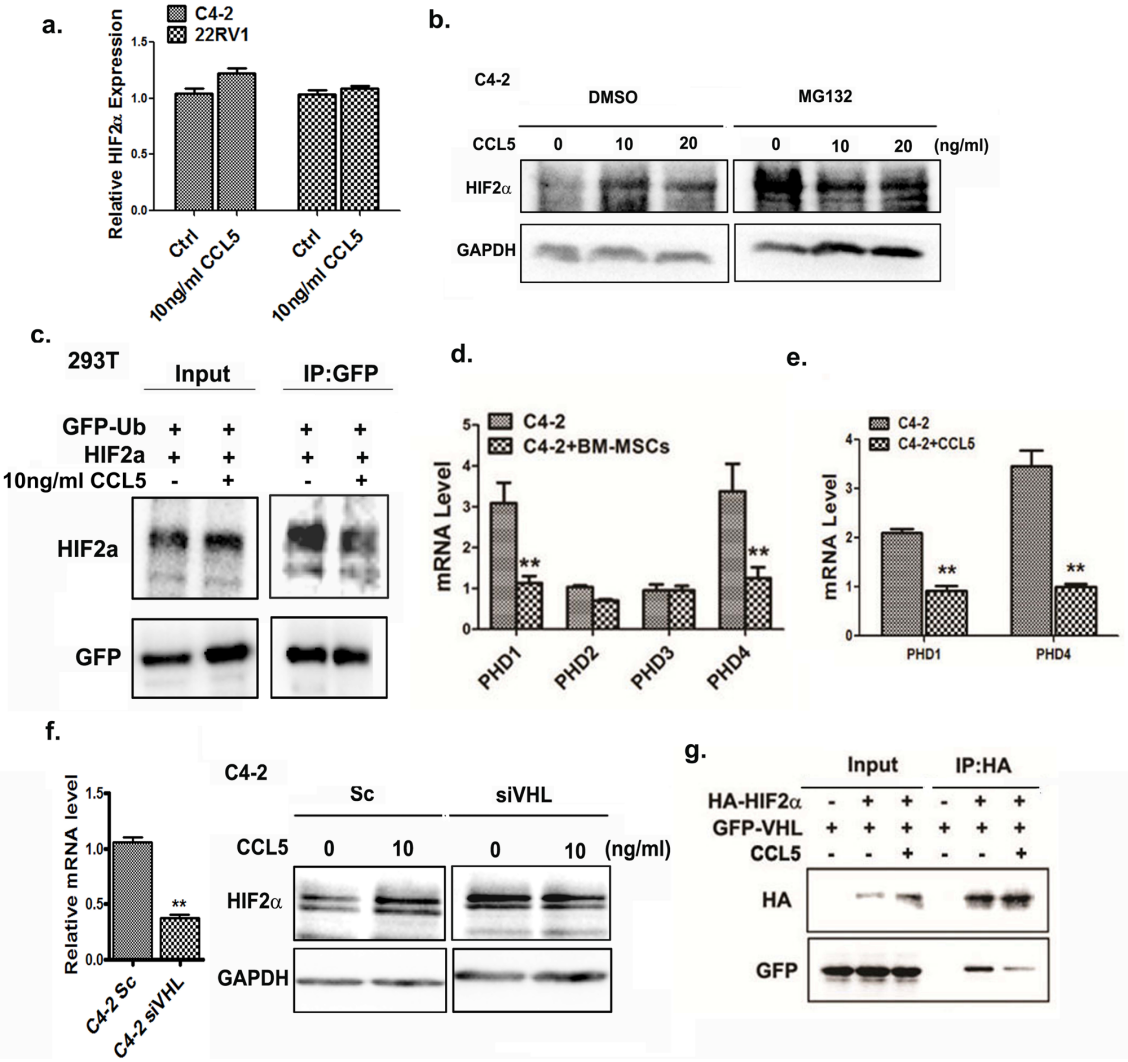
CCL5 suppresses the ubiquitination of HIF2α through VHL **a.** qPCR analysis of mRNA level of HIF2α in PCa cells after treating with CCL5. **b.** The Western blot analysis of HIF2α in C4-2 cells treated with different doses of CCL5 for 48 hrs, and then the DMSO or 20 μM MG132 were added into the cells for 4 hrs. The expression of HIF2α was analyzed. **c.** The ubiquitinaion assay of HIF2α in 293T cells. The 293T cells were transfected with the GFP-Ub and HIF2α plasmids. The GFP-Ub proteins were immuno-precipitated by GFP antibody, the HIF2α proteins were analyzed by Western blot. **d.** The PHD1/PHD4 expression was regulated by BM-MSCs. The C2-4 cells were co-cultured with BM-MSCs and mRNA expression of PHDs was investigated by qPCR analysis. **e.** qPCR analysis of PHD1 and PHD4 after incubating C4-2 cells with 10 ng/ml rCCL5 for 48 hrs. **f.** The expression of HIF2α in Sc/siVHL C4-2 cells after adding 10 ng/ml CCL5. Left panel showed that the VHL was successfully knocked down in C4-2 cells. **g.** Co-IP experiment. 293 cells were transfected with HIF2α and VHL, immunoprecipitated with HA antibody, and the VHL protein binding was detected in Western blot analysis using GFP antibody.

To further dissect the molecular mechanism(s) by which CCL5 increases HIF2α, we investigated whether CCL5 enhances the HIF2α mRNA expression. As shown in Figure 4a, addition of rCCL5 into the PCa cell culture could not increase the HIF2α at mRNA level significantly. Next, we tested whether CCL5 could regulate the stability of HIF2α protein. The C4-2 cells were treated with rCCL5, and the proteasome inhibitor MG132 or vehicle, and results revealed that MG132 blocked the CCL5-increased HIF2α expression (Figure 4b), suggesting that the CCL5 secreted by BM-MSCs may affect the protein stability of HIF2α in PCa cells.

We further investigated whether CCL5 can influence ubiquitination of HIF2α and the co-immunoprecipitation (CoIP) assay revealed that the CCL5 strongly inhibited the interaction between ubiquitin (Ub) and HIF2α. This suggests that the CCL5 may suppress the ubquitination of HIF2α. (Figure 4c) Next, we examined the expression of PHDs, members of the iron- and 2-oxoglutarate-dependent dioxygenase enzyme family [27], in PCa cells upon BM-MSCs co-culture, as early studies suggested the PHDs can catalyze hydroxylation of HIF2α and then promote its binding to VHL, a E3-ligase of the HIF2α [28]. It can be speculated that the HIF2α molecule may undergo degradation *via* proteasomal degradation after binding to VHL. Indeed, we found that expressions of the PHD1 and PHD4 molecules in PCa cells were dramatically decreased after co-culture with BM-MSCs (Figure 4d), and addition of rCCL5 into PCa cells suppressed the PHD1 and PHD4 expressions at mRNA levels (Figure 4e).

Since the PHDs can influence the ubiquitination of HIF2α by VHL E3-ligase [29], we further investigated whether the CCL5 mediated-HIF2α up-regulation is dependent on VHL. After knocking down the VHL in PCa cells by exploiting the VHL-siRNA, we observed that CCL5 no longer was able to mediate up-regulation of HIF2α in C4-2 cells (Figure 4f). We also examined whether CCL5 could influence the interaction of HIF2α and VHL molecules as early data showed that PHDs could hydroxylate the HIF2α to promote its interaction with the VHL [28]. We performed a CoIP assay *via* co-transfection of the HA-tagged HIF2α and GFP-VHL plasmids into the HEK293T cells in the absence and presence of rCCL5. After using the HA antibody to precipitate the HIF2α molecule, the co-precipitated VHL protein with the GFP antibody was visualized by Western blot analysis. As shown in Figure 4g, we found the interaction between HIF2α and VHL was significantly inhibited in the presence of rCCL5, suggesting that CCL5 suppressed the binding of HIF2α and VHL to prevent degradation of the HIF2α protein.

Together, results from Figure 4a–4g suggest that CCL5 could regulate the ubquitination of HIF2α *via* modulating the interaction between VHL and HIF2α.

### BM-MSCs and CCL5 induce HIF2α to suppress AR activity

**Figure 5:**
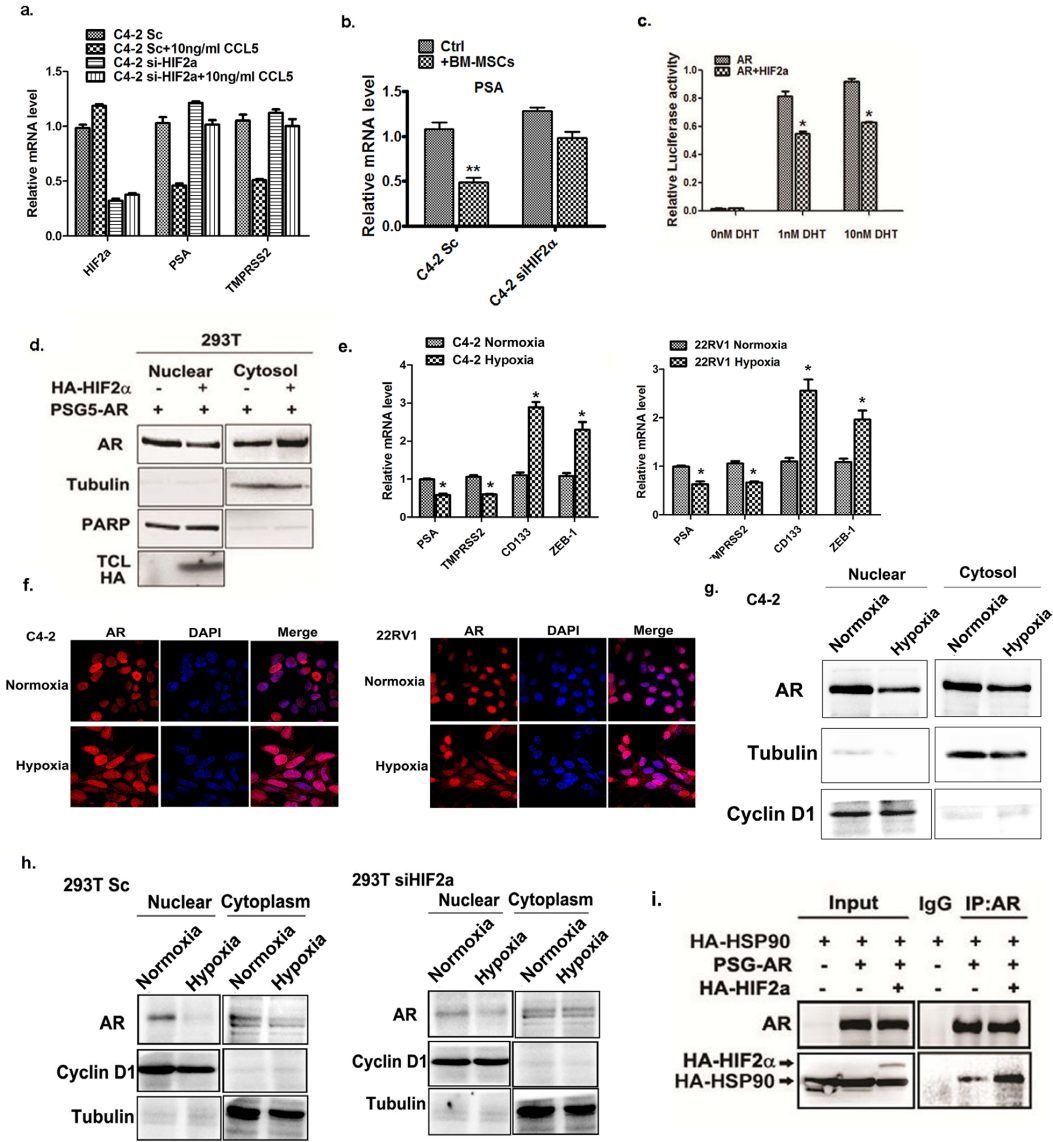
HIF2α suppress AR transactivation activity **a.** qPCR analysis of PSA, TMPRSS2 and HIF2α expression after treating C4-2 cells with CCL5. **b.** Sc or siHIF2α C4-2 cells were co-cultured with or without the BM-MSCs. The total RNAs were extracted and the expressions of the PSA genes were analyzed. **c.** HIF2α regulates AR signal. The luciferase assay was performed using MMTV-luc in 293 cells in the absence and presence of HIF2α. **d.** HIF2α regulates AR translocation. The 293 cells were transfected with the AR with or without HIF2α, the nuclear extraction were performed and detected by Western blot. Tubulin was used as the cytosol marker and the PARP was used as the nuclear marker. **e.** The qPCR analysis of PSA, TMPRSS2, CD133 and ZEB-1 expression in C4-2 and CWR22RV1 cells under normoxia and hypoxia (0.5% oxygen) condition. **f.** The immuno-fluorescent staining of AR under normoxia and hypoxia condition in C4-2 and CWR22RV1 cells. **g.** Hypoxia regulates the AR nuclear translocation. C4-2 cells were cultured under normoxia and hypoxia conditions, the nuclear extraction were performed and detected by Western blot. Tubulin was detected by Western blot as the cytosol marker; the cyclinD1 was detected as nuclear marker. **h.** Sc and siHIF2α 293T cells were cultured under normoxia or hypoxia condition. The AR localization was analyzed by western blot. **i.** The CoIP assay to detect the AR and HSP90 interaction was performed in 293 cells. Cells were transfected by HSP90, AR and HIF2α plasmids. The AR antibody was used for the IP of AR-HSP90 interaction complex and the pull-down protein complex was further detected by western blot.

Early studies indicated that BM-MSCs and CCL5 could promote PCa invasion *via* regulating AR activity [11]. AR has been identified as a key factor to influence PCa progression [13, 30–32], and recent studies also documented well that AR might suppress PCa metastasis [8, 9, 15, 26, 33]. We were interested to see if CCL5-HIF2α signals could function through altering the AR activity to increase the PCa cell invasion. We first knocked down the HIF2α in C4-2 cells by HIF2α-siRNA, and then added rCCL5 to examine the expression of AR downstream genes including PSA and TMPRSS2. The results revealed that rCCL5 treatment suppressed the expression of PSA and TMPRSS2 in C4-2 cells, and knocking-down the HIF2α interrupted the rCCL5 effect to suppress the expression of PSA and TMPRSS2 (Figure 5a). Knocking down HIF2a in PCa cells also suppressed the BM-MSCs-induced PSA expression (Figure 5b), suggesting that CCL5 might be able to modulate HIF2α signaling to influence AR activity. This conclusion was further demonstrated in the MMTV-ARE luciferase assay in HEK293T cells showing the addition of HIF2α led to suppress MMTV-ARE luciferase activity at 1 nM (human serum androgen concentration at castration stage) and 10 nM DHT (normal human serum androgen concentration) conditions (Figure 5c).

To further dissect the mechanism how HIF2α suppresses AR transactivation, we investigated whether HIF2α could influence AR nuclear translocation, the key step to activate AR [34, 35], and results revealed that addition of HIF2α suppressed the AR nuclear translocation (Figure 5d), suggesting that HIF2α might be able to suppress the AR transactivation *via* inhibiting the AR translocation from cytosol into nucleus.

Since hypoxia can induce the expression of HIF2α, we next investigated whether hypoxia also can regulate the AR activity. The C4-2 and CWR22RV1 cells were cultured at 0.5% oxygen and expression of the AR downstream genes were analyzed by qPCR. Interestingly, the cells under hypoxia condition showed lower expressions of AR downstream genes, while also showing higher expression of the cancer stem cell also markers. (Figure 5e) This result is consistent with the previous reports suggesting that HIF2α can increase cancer stem cell population and AR signaling is absent in cancer stem cells [26, 36].

Then we analyzed the AR localization under normoxia and hypoxia conditions. After culturing PCa cells under hypoxia condition for 24 hrs, we observed higher AR levels retained in the cytosol compared to the normoxia condition. (Figure 5f) The Western blot analysis also revealed that the AR translocation into the nuclear compartment in PCa cells was suppressed under hypoxia condition (Figure 5g).

We further knocked down the HIF2α in 293T cells, and treated cells with hypoxia. The results from Western blot analysis demonstrated that after knocking-down HIF2α, the hypoxia condition showed less effect to suppress the AR nuclear translocation. (Figure 5h), suggesting that HIF2α is necessary for hypoxia inhibition of AR nuclear translocation.

As the heat shock protein 90 (HSP90) is an important factor that regulates AR nuclear translocation *via* binding to cytosol AR to influence its nuclear translocation [37], we also examined the potential influence of HIF2α on the interaction of AR and HSP90. Using Co-IP assay, we found the interaction of these molecules was significantly increased upon HIF2α addition (Figure 5i), suggesting that HIF2α can suppress AR nuclear translocation *via* promoting the interaction of AR and HSP90 molecules.

Together, the results of Figure 5a–5i suggest that HIF2α suppressed the activation of AR *via* blocking the AR nuclear translocation process.

## DISCUSSION

**Figure 6:**
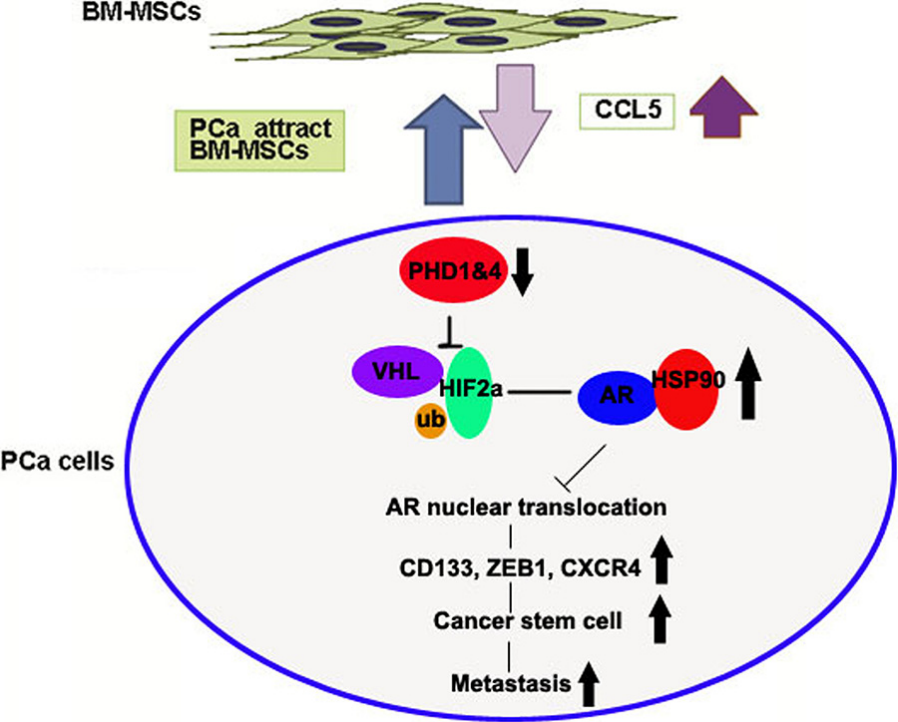
The cartoon of the pathway

Increasing numbers of reports suggested that BM-MSCs might represent an important component in the TME to influence the tumor growth and metastasis [4, 5, 38, 39]. An early study in breast cancer also suggested that CCL5 secreted from BM-MSCs might result in alteration of the cancer metastasis [38], yet the detailed mechanism involved in altering the downstream genes remained unclear. Here we report a new signaling from CCL5 secreted from BM-MSCs to increase the expression of HIF2α in PCa that resulted in suppression of AR transactivation. The consequences of such suppression might then enhance the PCa invasion. The linkage of CCL5-HIF2α signaling to AR-mediated PCa cell invasion described here might represent the first finding that provides us the opportunity to develop the potential new therapeutic approach to target this newly identified signaling to suppress the PCa metastasis (Figure 6).

The HIF family members are the key factors to respond to the hypoxia condition [40, 41]. They can promote PCa cell growth, angiogenesis and metastasis. [42] There are two isoforms (HIF1α and HIF2α) of the HIFα, and early studies were more focused on HIF1α roles in PCa [43]. While we also found HIF1α could be induced after recruited BM-MSCs (with increased CCL5) in PCa cells, we focused on HIF2α here as the recent studies showed that the HIF2α is more important for the cancer stem cell population induction [24, 25]. The PDHs are the most important regulators for the HIF protein stability; they can catalyze the hydroxylation of the HIF2α and then promote its binding to VHL, which is the E3-ligase of the HIF2α. These facts indicate that not only does low oxygen trigger the hypoxia system, but cytokines may also have the same effect.

While the AR has been extensively studied [30–32, 44] as a positive factor to promote PCa growth, and ADT to reduce or prevent androgens from binding to AR can effectively to suppress PCa cell growth during the first 12–24 months treatment, however, eventually most ADT fails and tumors re-grow with development of castration resistance [45–49]. However, more and more evidences with different approaches, suggested that AR might function as suppressor for the PCa metastasis. [9, 12, 13, 15, 26, 33]. Here we add evidence showing that infiltrating BM-MSCs can go through modulation of CCL5-HIF2α signaling to suppress AR transactivation that may then enhance PCa cell invasion. Therefore, the development of a new combination therapy of ADT plus anti-CCL5/HIF2α/AR signaling may help us to overcome the failure of ADT with better efficacy to suppress PCa cell invasion.

## MATERIALS AND METHODS

### Cell culture

LNCaP, C4-2, and CWR22RV1 cell lines were purchased from the American Type Culture Collection (ATCC, Manassas, VA) and cultured in RPMI 1640 with 10% FBS. Human BM-MSCs were purchased from Stemcell Technologies (Vancouver, BC) and cultured in Human MesenCult^®^ Proliferation Kit (Stemcell Technologies Inc). All cells were maintained in a humidified 5% CO_2_ environment at 37°C.

### Cell invasion assay

Six (0.4 μm pore size) or 24-well (8 μm pore size) transwell plates (Corning, Lowell, MA) were used for co-culture and invasion assay, respectively. PCa cells were co-cultured with BM-MSCs in transwell plates for 36–48 hrs. For *in vitro* invasion assays, transwell plate membranes were pre-coated with diluted matrigel (20%) (BD Biosciences, Sparks, MD) and PCa cells (10^5^ cells in serum free medium) were plated in the upper chambers while 10% serum containing media placed in the lower chambers. After 39–48 hrs incubation, cells invaded into the lower chambers and attached to the lower part of the membranes were stained with toluidine blue, and positively stained cells were counted. The cell numbers were counted in six random fields. Quantitation indicates means of triplicate repeats ± SEM.

### RNA extraction and quantitative real-time PCR (qPCR) analysis

Total RNAs were isolated using Trizol reagent (Invitrogen, Grand Island, NY). One μg of total RNA was subjected to reverse transcription using Superscript III transcriptase (Invitrogen, Grand Island, NY). qRT-PCR was conducted using a Bio-Rad CFX96 system with SYBR green to determine the mRNA expression level of a gene of interest. Expression levels were normalized to GAPDH level.

### Western blot analysis

Cells were lysed in RIPA buffer and proteins (20–40 μg) were separated on 8–10% SDS/PAGE gel and then transferred onto PVDF membranes (Millipore, Billerica, MA). After blocking membranes, they were incubated with primary antibodies, HRP-conjugated secondary antibodies, and visualized using ECL system (Thermo Fisher Scientific, Rochester, NY). AR, GAPDH, tubulin, PARP, Cyclin D1 and GFP antibodies were from Santa Cruz Biotechnology, Inc (Santa Cruz, CA). HIF2α antibody was purchased from Novus Biologicals (Littleton, CO) and Cell Signaling Technology (Beverly, MA)

### Histology and immunohistochemistry

Mouse tissues obtained obtained were fixed in 10% (v/v) formaldehyde in PBS, embedded in paraffin, and cut into 5-μm sections. Prostate tissue sections were deparaffinized in xylene solution and rehydrated and immunostaining was performed. HIF2α antibody was purchased from Novus Biologicals (Littleton, CO).

### Luciferase assay

PCa cells were plated in 24-well plates and transfected with MMTV-luc containing ARE sequence using Lipofectamine (Invitrogen, Grand Island, NY). After transfection, regular media were added with various DHT concentrations, 0 (ethanol as vehicle control), 1 nM, and 10 nM, and incubated for 48 hrs. pRL-TK was used as internal control. Luciferase activity was measured by Dual-Luciferase Assay (Promega, Madison, WI) according to the manufacturer’s manual.

### *In vivo* orthotopic mice injection

The nude mice were from the Jackson Lab (Bar Harbor, Maine). CWR22RV1 cells were engineered to express luciferase reporter gene (PCDNA3.0-luciferase) by stable transfection and the positive stable clones (luc-CWR22RV1) were selected and expanded in culture. 20 control group mice (6–8 wks) were injected with luc-CWR22RV1 cells (1 × 10^6^, mixed with Matrigel, 1:1) and 10 test group mice were co-injected with PCa cells with primary BM-MSCs (1 × 10^5^). After sacrificing mice at 6 wks, the tumors were analyzed by IHC staining.

### Statistics

The data values were presented as the mean ± SEM. Differences in mean values between two groups were analyzed by two-tailed Student’s *t* test. *p* ≤ 0.05 was considered statistically significant. *, *P* value < 0.05; **, *P* value < 0.005.

## CONCLUSION

Together, our studies suggest that recruited BM-MSCs can secrete CCL5 to suppress the PHDs expression to induce the HIF2α expression. The consequences of such induced HIF2α expression can lead to enhance the AR-HSP90 interaction to suppress the AR nuclear translocation and AR transactivation that results in promotion of the PCa metastasis.
